# A Re-Appraisal of Pathogenic Mechanisms Bridging Wet and Dry Age-Related Macular Degeneration Leads to Reconsider a Role for Phytochemicals

**DOI:** 10.3390/ijms21155563

**Published:** 2020-08-03

**Authors:** Roberto Pinelli, Francesca Biagioni, Fiona Limanaqi, Miorica Bertelli, Elena Scaffidi, Maico Polzella, Carla Letizia Busceti, Francesco Fornai

**Affiliations:** 1 SERI, Switzerland Eye Research Institute, Riva Paradiso 2, 6900 Lugano, Switzerland; pinelli@seri-lugano.ch (R.P.); medicale@seri-lugano.ch (M.B.); relazioniesterne@seri-lugano.ch (E.S.); 2 I.R.C.C.S. Neuromed, Via Atinense, 18, 86077 Pozzilli, Italy; francesca.biagioni@neuromed.it (F.B.); carla.busceti@neuromed.it (C.L.B.); 3 Department of Translational Research and New Technologies in Medicine and Surgery, University of Pisa, Via Roma 55, 56126 Pisa, Italy; f.limanaqi@studenti.unipi.it; 4 Aliveda Laboratories, Viale Karol Wojtyla 19, 56042 Pisa, Italy; maico@aliveda.com

**Keywords:** autophagy, proteasome, immunoproteasome, oxidative stress, inflammation, retinal pigment epithelium, retinopathy, lutein, resveratrol

## Abstract

Which pathogenic mechanisms underlie age-related macular degeneration (AMD)? Are they different for dry and wet variants, or do they stem from common metabolic alterations? Where shall we look for altered metabolism? Is it the inner choroid, or is it rather the choroid–retinal border? Again, since cell-clearing pathways are crucial to degrade altered proteins, which metabolic system is likely to be the most implicated, and in which cell type? Here we describe the unique clearing activity of the retinal pigment epithelium (RPE) and the relevant role of its autophagy machinery in removing altered debris, thus centering the RPE in the pathogenesis of AMD. The cell-clearing systems within the RPE may act as a kernel to regulate the redox homeostasis and the traffic of multiple proteins and organelles toward either the choroid border or the outer segments of photoreceptors. This is expected to cope with the polarity of various domains within RPE cells, with each one owning a specific metabolic activity. A defective clearance machinery may trigger unconventional solutions to avoid intracellular substrates’ accumulation through unconventional secretions. These components may be deposited between the RPE and Bruch’s membrane, thus generating the drusen, which remains the classic hallmark of AMD. These deposits may rather represent a witness of an abnormal RPE metabolism than a real pathogenic component. The empowerment of cell clearance, antioxidant, anti-inflammatory, and anti-angiogenic activity of the RPE by specific phytochemicals is here discussed.

## 1. Introduction

Age-related macular degeneration (AMD) is a multifactorial disease affecting the retina which progressively leads to loss of vision up to irreversible blindness among elderly people in Western countries [1,2,3,4]. The incidence of AMD is increasing dramatically over time; in fact, it is predicted to affect up to 288 million people worldwide by 2040 [5]. About 85% of individuals with AMD present with the “dry” (atrophic) variant, which is classically distinguished from the “wet” (exudative or neovascular) one, concerning pathology, time course, and severity [3,4,5,6]. Although the classic nosography distinguishes between these two AMD phenotypes, the dry form may slowly progress into the wet one, which otherwise may be already “wet at the onset” [7]. In these cases, the wet AMD emerges abruptly and rapidly progresses to blindness [4,8]. This is the final outcome also for the dry form, which, despite progressing at a slower rate, eventually impairs visual acuity up to blindness [9]. 

The term macular degeneration indicates a degenerative process of the retina at the level of the fovea, which impairs visual acuity, and it is characterized at the pathological level by the presence of drusen between the retinal pigment epithelium (RPE) and the Bruch’s membrane, as defined in the authoritative review papers by de Jong and Jager et al. [3,4]. Similar to drusen, “pseudo-drusen” or “subretinal drusenoid deposits” occurring between the RPE and the boundary between the inner and outer segments of the photoreceptors are associated with an increased risk of developing advanced AMD [10,11,12,13]. In AMD, extra-macular areas may be involved early on, while in later stages, a widespread degeneration often appears involving both macular and extra-macular regions, which is defined as “geographic atrophy” (GA) [14,15]. In this case, scattered and/or confluent areas of retinal degeneration are evident. From a pathological perspective, AMD should feature the presence of drusen and/or subretinal drusenoid deposits, along with alterations of the retinal pigment epithelium (RPE), Bruch’s membrane, and, depending on the isoform, the proliferation of choroid vessels [3,4,8,12,16,17,18]. This is the case of wet AMD, which is typified by abundant exudation and choroidal neovascularization (CNV), wherein newly immature blood vessels grow to trespass the choroid–retinal border. These newly formed overwhelming vessels lead to fluid accumulation and even bleeding in the extracellular space, which increases interstitial pressure, while leading to the development of fibrosis around the neovascular tufts [3,4,8]. This represents the key distinguishing point between dry and wet AMD, which rapidly and consistently worsens visual processing. Advanced AMD, including both the GA and/or CNV variants, is characterized by confluent atrophy and extensive loss of macular photoreceptors, which cannot provide anymore for visual acuity and contrast sensitivity [3,12,19]. This is often accompanied by lines distortion known as metamorphopsia. Thus, despite a clear-cut dichotomy between wet and dry AMD, an overlap exists both in the phenotypes and the biochemical mechanisms underlying these seemingly disparate clinical conditions [19,20]. In fact, wet AMD often occurs on a background of dry AMD, and both forms may concur at different stages of AMD, which is in line with observations suggesting that GA and CNV are different though interconnected manifestations of the same disease [19,21]. 

The present review is focused on the RPE as the anatomical site which takes a center stage in the development of both dry and wet AMD. The review hypothesis is centered primarily on the RPE, which triggers alterations involving the light-sensitive photoreceptors outer segments (POS) on the one side, and the Bruch’s membrane and the capillary vessels of the inner choroid on the other. In fact, the updated functional anatomy of the retina is compatible with the notion that RPE acts as a kernel in retinal homeostasis [20,22,23,24]. Thus, the cellular and metabolic responses of the RPE to age-related changes may be a key in the pathogenic mechanisms involved in AMD onset and progression [20,22,23,24]. As we shall see, these may include (i) a failure in RPE-dependent retinal protection from oxidative and mitochondrial stress; (ii) a loss of RPE ability to cope with lipid, glycogen, and protein overload; (iii) impaired renewal by RPE of POS; (iv) a loss of the outer blood–retinal barrier (oBRB), which is mostly provided by RPE; (v) the occurrence of abnormal inflammatory/immune response; (vi) a loss of RPE polarity, thus affecting the metabolic flow of nutrients, growth factors, photoreceptor segments from the choroid to photoreceptors and vice versa; and (vii) accumulation of extracellular waste material, which brings the classic pathological hallmark of AMD represented by drusen (amorphous proteinaceous material including advanced glycation end products (AGEs) and lipids). In this frame, it is remarkable that the metabolic-, oxidative-, and inflammatory-related pathways being activated within the aged/stressed RPE do engage the cell-clearing systems autophagy and proteasome, which are critical to ensure retinal functions, while preventing neurodegeneration. In fact, autophagy and proteasome are altered in both AMD patients and experimental models, and inhibition of either autophagy or proteasome in experimental models reproduces key pathological features of both dry and wet human AMD [25,26,27,28,29,30,31,32,33,34,35,36,37,38,39,40,41,42,43,44].

Nonetheless, a deep knowledge of AMD pathogenic mechanisms is still lacking so far, which complicates the establishment or approval of effective treatment strategies. While the overactive angiogenesis and visual deficits occurring in wet AMD can be mitigated by anti-VEGF drugs, this is not the case for dry AMD [45] Recent evidence-based studies show that dietary phytochemicals such as lutein/zeaxanthin, resveratrol, and *Vaccinium myrtillus* may be beneficial in retinal diseases including AMD [46,47,48,49,50,51,52,53,54,55,56]. In general, these compounds have been claimed to interfere with the biology of disease by mitigating oxidative stress and inflammation, which play a critical role in the initiation and progression of AMD [47,53,54,55,56]. However, some pieces of evidence suggest that, besides oxidative stress and inflammation, the beneficial effects of these phytochemicals may be explained by a potential involvement in cell-clearing system alterations within RPE, which were briefly summarized so far as candidate pathogenic events to produce retinal damage in AMD [56,57,58,59,60]. Thus, a synergism between these compounds may extend to cell clearing pathways, which play a fundamental role in tuning the orchestration at the retinal-choroid border, where RPE cells may be the pivot. 

In light of these considerations and in the frame of the metabolic, oxidative, and inflammatory mechanisms operating in AMD, the present review is an attempt to bridge macular degeneration, cell-clearing systems, and phytochemicals. A special emphasis is put on the RPE as the seat where cell-clearing systems are expected to be more critical. In fact, advancement in the functional anatomy of the outer retina allows us to better comprehend the role of this region in the specific steps implicated in retinal integrity or degeneration. 

## 2. Cell-Clearing Systems in the RPE as the Keys for Retinal Integrity 

### 2.1. RPE Anatomy and Physiology

With its apical microvilli-enriched membrane, the RPE faces the extracellular matrix of the subretinal space, which enables its interaction with the POS through relatively weak adhesive forces [61,62]. Through its basolateral membrane, the RPE faces the Bruch’s membrane, an extracellular matrix which acts as an anatomical barrier and molecular sieve between the retina and the blood flow in the fenestrated vessels of the choriocapillaris [63]. The RPE also displays an inherent polarity, with a basolateral side of tight junction-connected cells creating a barrier for the choriocapillaris. This makes RPE cells protectors and key effectors of the outer blood–retinal barrier (oBRB), which, among many functions, grants retinal immune-privilege [62,64]. Transport-related organelles are preferentially placed in the basal cytoplasm of the RPE, while melanosomes, the light-absorbing pigments, are primarily located at the apical level [62]. These unique features configure the RPE as a kernel in tuning retinal homeostasis through mutual interactions between the RPE and either the photoreceptors or the choroid. In fact, the RPE carries out several retinal functions, such as light absorption, visual cycle, POS phagocytosis and renewal, immune modulation, and epithelial transport and secretion [61,62,64]. The RPE serves as a source of polarized growth factor release and transporter of ions, water, and metabolic waste products from the subretinal space to the blood, while delivering growth factors and blood-derived nutrients to the photoreceptors. For instance, from the apical side, RPE cells release pigment epithelium-derived factor (PEDF), which promotes photoreceptor survival, while exerting an antiangiogenic effect on the choriocapillaris [65]. Similarly, neuroprotectin D1, an anti-inflammatory and cell survival factor derived from docosahexaenoic acid (DHA), and αB crystalline, a chaperone protein with anti-apoptotic and anti-inflammatory functions, are released from the apical side of RPE cells toward either photoreceptors or adjacent RPE cells [66,67]. Instead, the vascular endothelial growth factor (VEGF) is secreted in low concentrations on the basolateral side, to ensure the development and maintenance of the choriocapillaris [68,69].

While fulfilling the high metabolic demands of the retinal milieu, the RPE also maintains its structural integrity through an efficient defense against free radicals, photo-oxidative exposure, and light energy, which physiologically occur during the visual cycle [61,62]. The RPE is the site where retinoic acid coming from blood vessels is converted into its aldehyde form (11-*cis* retinal) [70]. In this way, the 11-*cis* retinal may be provided to the photoreceptors. In fact, the 11-*cis* retinal exits the RPE, and through the subretinal space, it enters the POS, where it combines with opsin ready to be converted by light into all-*trans* retinal [70]. All-*trans* retinal is then released from the opsin and can be transported to the RPE, which can convert it back to 11-*cis* retinal. In this way, the RPE maintains the supply of 11-*cis* retinal chromophore for the regeneration of photo-oxidized visual pigments. Again, the RPE supports vision by granting the daily degradation and renewal of photo-oxidized POS, which are rich in polyunsaturated fatty acids, opsins, retinals, and *bis*-retinoids all deriving from the photo-transduction signaling cascade, along with other components of the POS [71]. 

The RPE also absorbs light through melanin granules, which are synthesized by the catalysis of tyrosine to L-DOPA [72]. Besides serving as an absorbing pigment, melanin also quenches singlet oxygen, it scavenges reactive radical species, and it chelates metals [72,73,74]. Melanin synthesis diminishes with age, and the constant exposure of the RPE to high levels of oxygen and light may occlude its antioxidant properties [72]. Remarkably, RPE cells also express L-dopa decarboxylase (DDC), thus being able to synthesize the neurotransmitter dopamine (DA) [75]. Both L-DOPA and mostly DA may undergo self-oxidation to produce highly oxidative species [73,76]. An altered L-DOPA metabolism shifting toward DA synthesis may contribute to the generation of cytotoxic, nitrogen, and oxygen reactive species within RPE cells [73]. Such a load of free radicals within the RPE is expected to add on the high photo-oxidative environment and to the oxygen overflow deriving from the photoreceptors and blood side, respectively. In a way which is reminiscent of the high susceptibility of DA neurons in Parkinson’s disease (PD), this may explain why the RPE is highly susceptible to age-related mitochondrial and oxidative damage, fostering the accumulation of oxidized lipofuscin, an auto-fluorescent heterogeneous mixture of lipid–protein aggregates [24,30,77]. In this frame, to our knowledge, no study so far investigated within the RPE the presence of monoamine oxidase (MAO), which may provide a strong protective effect in counteracting DA self-oxidation [78]. This may contribute to explain the high prevalence of AMD being potentially the sole condition in which DA synthesis occurs in the absence of MAO. In fact, it is well established that, when high intracellular DA levels are produced with impairment of MAO, strong protein oxidation takes place [76,79]. This is based mostly on the binding of self-oxidized DA to cysteinyl–protein residues [79,80]. 

The cellular and metabolic responses of the RPE to age-related changes are widely believed to mediate the pathologic processes involved in AMD onset and progression. Key metabolic pathways that orchestrate the RPE responses to various stressful conditions are autophagy and proteasome, the two major proteolytic systems being promiscuously involved in the removal of damaged proteins and organelles [32,44,81,82,83,84]. A decline in autophagy and proteasome activity occurs with aging and key pathogenic mechanisms that are implicated in the development and progression of AMD, including chronic oxidative stress, inflammation, and drusen, are bound to alterations of autophagy and proteasome activities [25,26,27,28,29,30,31,32,33,34,35,36,37,38,39,40,41,42,43,44,82,85,86]. 

In the following sections, we discuss the intersection of autophagy and proteasome system with RPE homeostasis, and the oxidative and inflammatory mechanisms that are implicated in AMD. 

### 2.2. Cell-Clearing Systems Coping with Oxidative Stress and Inflammation in the RPE

**Figure 1:**
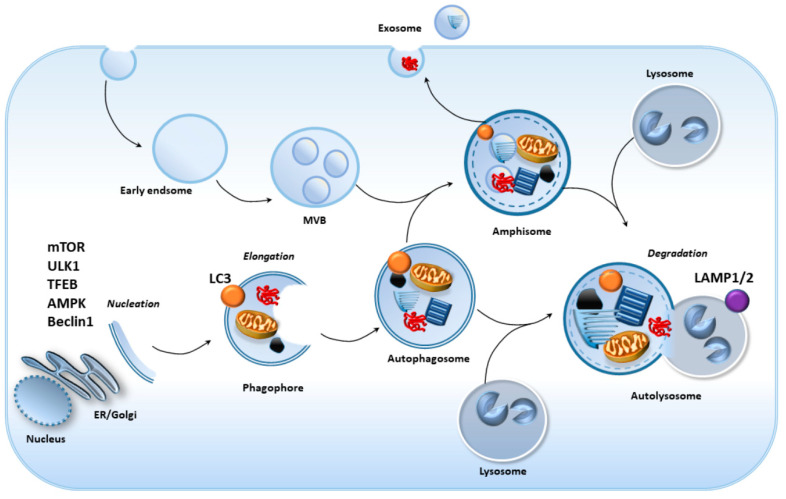
Schematic overview of the autophagy machinery progression. The recruitment of Atg proteins to the phagophore assembly site (nucleation) is followed by the formation of the phagophore, which engulfs various intracellular substrates. The phagosome gives birth to the autophagosome, which may either fuse with late endosomes (multivesicular bodies, MVB), leading to the formation of the amphisome, or fuse directly with lysosomes. The autolysosome, where cargo degradation eventually occurs, derives from the fusion of the amphisome or autophagosome with the lysosome. When amphisomes do not fuse with lysosomes, partially indigested cargos can be spread extracellularly via exosomes.

Autophagy is a tightly regulated cell-clearing machinery which proceeds through several steps to degrade intracellular substrates and grant cell homeostasis (Figure 1). Autophagy initiation is regulated by several molecular pathways that may act either in concert or independently of each other, including mammalian/mechanistic target of rapamycin kinase (mTOR), Unc-51 Like Autophagy Activating Kinase 1 (ULK1), 5’ AMP-activated protein kinase (AMPK), transcription factor EB (TFEB), and Beclin 1. Autophagy initiation goes along with the recruitment of Atg proteins to the phagophore assembly site (nucleation) and for the formation of the phagophore (elongation) (Figure 1). The phagophore engulfs various intracellular substrates, including lipids, glycogen, proteins, and whole organelles. The phagosome then seals and matures to give birth to the autophagosome, which may either fuse with late endosomes (multivesicular bodies, MVB), leading to the formation of the amphisome or directly with lysosomes. The fusion of the amphisome or autophagosome with the lysosome gives birth to the autolysosome, where cargo degradation eventually occurs. When amphisomes do not fuse with lysosomes, partially indigested cargos can be spread extracellularly via exosomes.

In the retina, autophagy is coupled with the circadian rhythms. In fact, it exhibits a bimodal pattern that correlates with shifting in transduction within photoreceptors and circadian rhythm of POS phagocytosis in the RPE [87]. In healthy mice kept under normal cycling light conditions, the shifts between light and dark translate into a sharp decrease, followed by a time-dependent increase in autophagy within photoreceptor cells. In detail, the translocation of transducin and arrestin from the outer to the inner segment of photoreceptors contributes to a light-dependent upregulation of autophagy [87]. These findings back up pioneering studies showing that autophagy-dependent digestion of opsin or rhodopsin within photoreceptors is necessary for adaptation to abruptly increased habitat illuminance through the removal of surplus visual pigment [88]. In turn, the cyclic variations of autophagy within the RPE are tuned by the circadian ingestion of POS [87]. In baseline conditions, autophagy within RPE cells is induced by all-trans-retinal, while upon sustained oxidative stress and light exposure, impairment of autophagy and mitophagy is detected, which goes along with delayed all-trans-retinal clearance [36]. Such a circadian autophagy rhythm is critical since mice lacking Beclin-1 or Atg7 develop severe retinal degeneration upon light exposure [36]. Similarly, a phase-shifting of autophagy proteins occurs in experimental retinopathy, which remarkably impairs autophagy itself [89]. The critical role of autophagy within RPE is emphasized in mice models where the lack of Atg5 or Atg7 focally within RPE cells leads to retinal degeneration compatible with AMD-like phenotypes [33].

**Figure 2:**
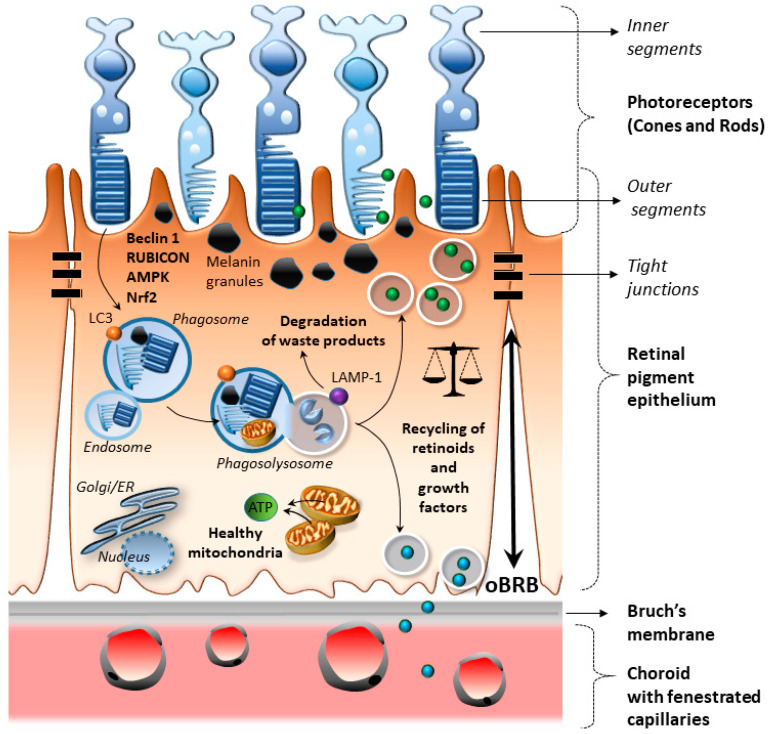
Autophagy grants retinal pigment epithelium (RPE) functions and homeostasis. Within the RPE, Beclin 1/Rubicon, AMPK, and Nrf2 trigger heterophagy, which is the concerted action of LC3-associated autophagy and phagocytosis, which is crucial for the degradation and renewal of the outer segments of photoreceptors. A functional autophagy flux within RPE cells also grants redox and energy homeostasis by degrading altered mitochondria and oxidized material, including melanin granules, lipids, and lipofuscin. At the same time, some metabolic by-products, including retinoids and growth factors, are delivered to the photoreceptors or the inner choroid. A functional autophagy status goes along with the maintenance of the outer retinal–blood barrier integrity and a balanced polarity of RPE cells.

Within the RPE, autophagy is a key in the visual cycle, since it tunes the removal of the distal tips of the photoreceptors while recycling metabolic by-products and growth factors to be delivered back to photoreceptors [82] (Figure 2). In detail, the degradation of POS occurs through a concerted effort between phagocytosis and non-canonical LC3-related autophagy, which is also known as heterophagy [82,90]. In detail, the morning burst of RPE phagocytosis matches with the enzymatic conversion of the autophagy protein LC3 to its lipidated analog LC3-II, which is associated with non-canonical single membrane phagosomes containing engulfed POS [90]. This occurs in an Atg5-dependent manner that requires Beclin1 but not the autophagy pre-initiation complex ULK1. In fact, mice with Atg5-deficient RPE cells feature disrupted lysosomal processing of POS, decreased photoreceptor responses to light stimuli, and decreased chromophore levels [90]. RPE cells are able to finely tune autophagy dynamics through several mechanisms during the critical period of POS phagocytosis [91]. In the morning, when POS phagocytosis occurs, RPE cells activate autophagy through RUN domain and cysteine-rich domain-containing Beclin 1-interacting protein (RUBCN/Rubicon). Once POS phagocytosis occurs, RPE cells suppress autophagy through the activation of the epidermal growth factor receptor (EGFR) and mammalian/mechanistic target of rapamycin kinase (mTOR) [91]. This goes along with the accumulation of SQSTM1/p62 and the phosphorylation of Beclin 1 (BECN1) on an inhibitory residue occluding autophagy. Recent studies show that activation of nuclear factor erythroid 2-related factor 2 (Nrf2) and 5’ AMP-activated protein kinase (AMPK) also contribute to the maintenance of RPE physiology via LC3-associated POS phagocytosis [31]. Thus, the physiological coupling between autophagy and diurnal rhythm appears as a key mechanism in the visual cycle. 

Two regulatory proteins of heterophagy-dependent POS degradation within RPE cells are βA3/A1-crystallin and melanoregulin proteins [92,93,94,95,96]. In detail, the loss of βA3/A1 decreases endo-lysosomal acidification eventually impairing POS degradation by heterophagy, as shown by POS being retained at the basal side of RPE cells [92,93]. This is associated with the inactivation of transcription factor EB (TFEB), mTOR stimulation, and a decrease in the levels of cathepsin D, which eventually impairs endosome-autophagy-lysosomal function and the proteolysis of POS and rhodopsin within RPE phagosomes [92,93,97]. A similar role was recently reported for another member of crystallin family proteins, the αB co-chaperone, which, when mutated, inhibits fusion of autophagosomes with lysosomes within RPE cells [98]. On the other hand, the loss of melanoregulin, while altering retrograde melanosome transport in melanocytes, leads to accumulation of phagosomes and lipofuscin in the RPE, as well as abnormal cathepsin D secretion, which produces a slowly progressive retinal damage affecting the RPE and spreading to internal layers [94,95,96,99]. This is reminiscent of what occurs in AMD, which involves the RPE extending to internal and external layers. Remarkably, circadian variations in melanoregulin expression in the RPE are closely associated with the Atg5-dependent non-canonical LC3-related autophagy, with melanoregulin coordinating the association of LC3 with phagosomes [95].

In line with this, autophagy is implicated in melanin metabolism by tuning melanolysis and melanogenesis [100,101,102]. Suppression of Atg7-dependent autophagy inhibits melanogenesis and promotes oxidative-stress-induced apoptosis of melanocytes [102]. Rescuing autophagy enhances melanocyte proliferation and protects from oxidative stress by downregulating reactive oxygen species (ROS) through Nrf2 activation. This may be the key in RPE cells, where redox homeostasis is crucial for the light-absorbing and antioxidant biological functions of melanin. Sustained retinal oxidative stress during aging may decrease melanin content, while reducing its antioxidant capacity [42,103]. Moreover, increased melanosomal oxygen consumption and ROS production may foster melanosomal–lipofuscin accumulation, which may be detrimental for RPE cells when clearing systems are impaired [42,104,105].

#### 2.2.1. Oxidative Stress

Autophagy is a key to coping with oxidative stress, which is constantly faced by the RPE and represents a key factor in the development of AMD [25,28]. In fact, an autophagy dysfunction in AMD-RPE cells is associated with increased susceptibility to chronic oxidative stress [24,28]. This is also bound to the accumulation of damaged mitochondria and a subsequent metabolic shift from oxidative phosphorylation to glycolysis [28]. In fact, a decrease in ATP production by mitochondria going along with an increased ATP production by glycolysis occurs in AMD-RPE cells featuring an autophagy dysfunction compared with normal RPE cells [28]. While maintaining energy homeostasis during the early stages of RPE injury, this is likely to elicit a dysfunction of RPE cells, which cannot cope with high levels of energy production from glycolysis during terminal stages [28]. Thus, in oxidative-challenged RPE, dysfunctional autophagy, being bound to a loss of Nrf2 and PCG1α, fails to orchestrate mitophagy and mitochondriogenesis, further promoting mitochondrial disintegration and a vicious cycle of oxidative-stress-related events up to caspase-mediated apoptosis [30,106,107]. In fact, mitochondrial alterations promote a highly oxidant intracellular milieu, which in turn affects lipid and glucose metabolism leading to the accumulation of oxidized lipids, proteins, and glycogen. In line with this, autophagy is the key to counteracting the accumulation of lipid droplets and glycogen granules, which occurs in AMD-RPE cells similar to other neurodegenerative conditions [28,108,109,110]. These events may add to the impaired digestion of POS-derived oxidized PUFAs and bis-retinoids in aged RPE cells, eventually promoting lipofuscin accumulation, which further sensitizes RPE cells to light-induced oxidative stress and protein misfolding [31,37,111]. Upregulation of autophagy markers and heat-shock proteins (HSPs) has been detected in RPE from both human AMD donors and rodent models [26,106,112]. On the one hand, this indicates the occurrence of an early, compensatory attempt to cope with increasing protein overload in the aging RPE cells; in fact, age-related cellular stresses, such as intense light, acute oxidative stress, mitochondrial alterations, and para-inflammation, may promptly recruit autophagy to grant cell survival [25,90]. Compensatory activation of autophagy may also occur following proteasome impairment that manifests in various neurodegenerative disorders and AMD, likely due to an oxidative-related disassembling of proteasome subunits and a decrease in its catalytic capacity [39,81,84,112,113,114]. 

**Figure 3:**
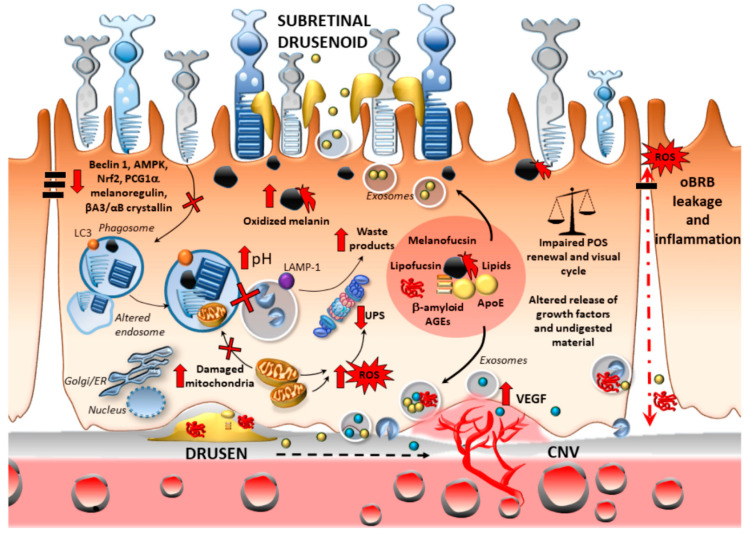
Autophagy and proteasome failure within RPE cells may foster AMD onset and progression. In aged RPE cells, impaired heterophagy due to the downregulation of Beclin 1, AMPK, Nrf2/PGC1a, melanoregulain, and crystallin co-chaperones occludes the digestion of the outer segments of photoreceptors, thus promoting the accumulation of damaged mitochondria and oxidized substrates such as lipofuscin and melanin. This further sensitizes RPE cells to light-induced oxidative stress, proteasome impairment, and protein misfolding. This goes along with an altered polarity of RPE cells due to abnormal, exosomal secretion of waste products (melano-lipofuscin, AGEs, oxidized lipids, and beta-amyloid), growth factors (VEGF and crystallins), and cathepsin D at either RPE sides. These events eventually contribute to impairing the visual cycle and photoreceptor’s metabolism, while promoting the formation of drusen and/or subretinal drusenoid deposit, the leakage of the outer blood–retinal barrier (oRBR), the recruitment of inflammatory mediators, and, eventually, CNV and angiogenesis fostering transition from dry to wet AMD.

As recently emphasized, a coordinated, either compensatory or synergistic, interplay occurs between autophagy and proteasome at several molecular levels, and the two systems may also converge within a single organelle named “autophagoproteasome” [83,84,115]. This has been extensively reviewed elsewhere and is not be dealt with here [84]. However, we wish to point out that, under chronic oxidative stress occurring in AMD, autophagy and the proteasome appear to be simultaneously affected (Figure 3). 

In fact, during sustained oxidative stress and proteasome impairment, an accumulation of altered mitochondrial and oxidation-prone lipofuscin occurs, which increases lysosomal pH and eventually occludes the autophagy flux affecting RPE cells, POS phagocytosis, and photoreceptor function [25,37,81,85,93,111,116,117]. In fact, similar to chloroquine, the major component of lipofuscin, the bis-retinoid N-retinylidene-N-retinylethanolamine (A2E), increases late endosomal/amphisomal and lysosomal pH, eventually impairing autophagy flux, while promoting lipofuscin accumulation [105,117] (Figure 3). Intriguingly, approaches promoting POS degradation through lysosomes re-acidification within impaired RPE cells include stimulation of beta-2 adrenergic, A2A adenosine, and D5 dopamine receptors [118]. This is interesting since adenosine, norepinephrine, and dopamine, which play a role in retinal function and POS phagocytosis [118,119,120,121,122], are known to variously affect mTOR-, AMPK-, and TFEB-related autophagy and also the proteasome system through stimulation of beta-adrenergic, A2A adenosine, and D1/D2-like dopamine receptors [123,124,125,126,127,128,129]. Since autophagy–lysosomal dysfunction associated with either an increased activity of mTORC1 or decreased activity of AMPK or TFEB does predispose to RPE and photoreceptors degeneration in experimental models [36,97,129,130,131,132], it would be worth investigating whether a coupling between alterations of autophagy activity and catecholamine innervation occurs in the frame of AMD. 

According to a current stream of evidence, the massive increase in autophagosomes and autophagy markers observed in RPE-AMD rather witnesses for a severe impairment of the autophagy flux, which is indeed detected in both human tissues from AMD donors and AMD experimental models [24,28,34]. Remarkably, AMD-RPE cells fail to induce autophagy in response to starvation [28]. Similar findings are observed within RPE cells of AMD rat models, where a decreased reactivity of autophagy occurs in response to fasting; conversely, occluding autophagy flux promptly exacerbates AMD-like pathological changes [34]. These findings suggest that an impairment of baseline RPE autophagy may be an early factor fostering AMD onset and progression. This is further substantiated by evidence in animal models, where autophagy inhibition produces a condition that is reminiscent of AMD [33,35]. This includes (i) oxidative and mitochondrial damage; (ii) accumulation of lipofuscin within RPE cells; (iii) disruption of RPE cells’ tight junctions; (iv) formation of drusen; (v) abnormal microglial activation; and (vi) release of pro-inflammatory and angiogenetic factors, up to CNV, and RPE and photoreceptors degeneration [33,35]. Again, this recalls the DA-containing cells of the substantia nigra, where autophagy inhibition reproduces PD, while rescuing autophagy grants the survival of DA-containing neurons [133,134,135]. In fact, just like RPE cells that produce DA, SNpc cells are susceptible to DA-related oxidative damage being bound to autophagy failure [115,135,136].

#### 2.2.2. Oxidative Stress and Outer Blood–Retinal Barrier Integrity

Chronic oxidative stress going along with autophagy dysfunction is implicated in RPE cell tight-junction disruption, eventually leading to oBRB leakage [137,138]. For instance, white light-emitting diodes (LED) featuring a high content of blue light cause structural alterations within the RPE, leading to the disruption of the oBRB, which is associated with an increase in oxidized substrates and impairment of basal autophagy [137]. Autophagy is also involved in selective degradation of the serine protease granzyme B [139], which is known to cleave RPE tight junctions and extracellular matrix (ECM) proteins, eventually contributing to a breakdown of the BRB and remodeling of the Bruch’s membrane. This is expected to consistently affect RPE-dependent transport between the retina and choroidal blood supply [140]. In line with this, granzyme B is increased in the RPE and choroidal mast cells in old, compared with young, donor eyes, and also in CNV patients’ eyes [140]. Again, in RPE cells, downregulation of thioredoxin-interacting protein (TXNIP) expression occurs under oxidative stress, which is associated with a block of the autophagy flux, leading to a decrease of RPE cell proliferation, disruption of RPE cell tight junctions, and a loss of oBRB integrity [138]. Remarkably, TXNIP knockdown, through upregulation of hypoxia-inducible factor 1-alpha (HIF-1α), enhances the secretion of VEGF from RPE cells, while stimulating angiogenesis in human retinal microvascular endothelial cells. Thus, by surveilling the oBRB within RPE cells, autophagy appears as a key mechanism keeping the choroidal vascular response from invading the retina and changing dry into wet AMD [64,138]. These findings suggest that an impairment of autophagy during sustained oxidative stress within RPE cells may promote CNV through VEGF-secretion-induced angiogenesis [138]. In fact, while impaired autophagy may promote angiogenesis by increasing the secretion of VEGFR2-containing exosomes [141] (Figure 3), rescuing autophagy may counteract the loss of oBRB integrity and abnormal VEGF secretion [142,143,144].

#### 2.2.3. Inflammation 

Chronic oxidative stress and/or the destruction of oBRB promote a local inflammatory response within the retinal milieu, which includes the release of danger-associated molecular patterns (DAMPs), NF-kB and inflammasome activation, abnormalities of the complement system, and the recruitment of immune cells and pro-inflammatory cytokines [20,27,44,86,145]. Besides dry AMD, this may be particularly relevant for CNV pathogenesis since the loss of oBRB integrity may potentiate the release of pro-inflammatory mediators adding to VEGF-induced CNV [146]. In detail, damaged RPE release PAMPs/DAMPs, which activate pattern recognition receptors in host cells, including Toll-like receptors (TLRs) and advanced glycosylation end product (AGE) receptors (RAGE) [44,86,147]. In fact, in advanced AMD, TLRs and RAGEs become highly expressed in RPE cells besides photoreceptors and choriocapillaris [148,149]. The binding of these receptors to their ligands leads to NF-κB, JAK/STAT, and NLPR3 inflammasome activation in neighboring cells, which may trigger apoptotic cell death, while further promoting the release of pro-inflammatory mediators [44,86,149,150]. 

Autophagy and proteasome are promiscuously implicated in the inflammatory events being triggered by these receptors. Since AGEs are physiologically cleared by autophagy and proteasome, occluding autophagy/proteasome activity promotes AGEs extracellular release and their binding to RAGE in neighboring cells [84,151,152,153]. Intriguingly, high levels of RAGE ligands, including oxidized low-density lipoproteins (oxLDL) and AGEs, are detected in extracellular drusen besides intracellular lipofuscin, suggesting a primary role of cell-clearing systems dysfunction in triggering unconventional secretions, which may foster drusen accumulation [148,154,155]. Again, autophagy controls NLRP3 inflammasome activation by degrading inflammasome components and effector molecules [43,156,157,158,159]. An autophagy decline going along with inflammasome overactivation may lead to RPE cell damage, tissue injury, and enhanced angiogenesis [27,43,160]. In RPE cells, inhibition of the proteasome, similar to autophagy, leads to NLRP3 release, IL-1β production, and caspase-1 activation, which is associated with a compensatory over-activation of HSP90 [43]. In fact, HSP90 inhibition prevents inflammasome over-activation in human RPE cells, while promoting NLRP3 degradation by autophagy [43].

A2E oxidation products, which are lipofuscin precursors and a further substrate of autophagy, are involved in complement activation and inflammation [161,162]. As part of the inflammatory response, complement activation can have beneficial effects by facilitating phagocytosis and removal of cellular debris; however, the complement cascade, and the alternative pathway, in particular, can be detrimental by causing bystander damage to surrounding tissues, which is associated with drusen and other sub-RPE deposits, and, in any case, vision loss in late AMD [45,145]. Complement alterations are also bound to dysfunctions of the endosomal–autophagy and proteasome systems [40,163]. In fact, in models of macular degeneration, early endosomes are abnormally enlarged, and they foster an increase of C3a fragments within RPE cells. This in turn leads to mTOR over-activation, promoting autophagy failure [163]. Similarly, C3a produced after C3 activation inhibits the proteasome within RPE cells, which is associated with impaired ECM turnover due to increased matrix metalloproteinase-2 (MMP-2) activity and formation of drusen-like deposits [40].

In this scenario, it is intriguing that apoptotic- and inflammatory-related intracellular pathways operating in AMD, including TLR/RAGEs, PKC, NF-kB, JAK/STAT, AKT/mTOR, and C3 complement, while impinging on the autophagy machinery and standard proteasomes, engage an alternative cytokine-inducible proteasome isoform, the immunoproteasome [38,84,127,164,165,166,167,168,169,170]. Contrarily from the standard proteasome, which is ubiquitously expressed, the immunoproteasome operates constitutively in immune tissues and cells, while being induced by oxidative stress and inflammatory cytokines in other kinds of cells, including neurons and glia of the retina and CNS [164]. The immunoproteasome possesses peculiar structural features and enhanced chymotrypsin-like catalytic activity compared with standard proteasomes [171]. These features allow the immunoproteasome to effectively process both oxidized/misfolded proteins and endogenous (self and viral) antigen peptides, leading to activation of CD8+ T-cell-dependent adaptive response via MHC-I presentation at the plasma membrane [164,171,172]. Despite a decline in proteasome activity being described in AMD, increased immunoproteasome expression has also been observed in the retina of AMD donors [173]. Again, in a mouse model of age-related RPE atrophy being deficient for monocyte chemoattractant protein-1, an upregulation of immunoproteasome subunits occurs, likely due to C3a complement-driven posttranslational alterations [38]. However, whether the immunoproteasome is detrimental or protective in the course of AMD pathogenesis appears controversial. For instance, the increase in β5i immunoproteasome subunits has been associated with RPE-mediated ECM abnormalities. These include a decrease in tissue inhibitor of metalloproteinases-1 (TIMP-1), along with an increase in MMP-2 and fibrosis-associated factors [38]. The immunoproteasome also promotes angiotensin-II-induced retinopathy through activation of the angiotensin II receptor type I (AT1R)-mediated signals [174,175]. This is associated with increased vascular permeability, oxidative stress, and NF-kB-mediated inflammation, which go along with impaired autophagy [174]. Again, an excessive proteasome activation due to increased STAT3 activation downstream of inflammatory signals is associated with the reduction of rhodopsin, decreased light reception, and photoreceptor cell function [176]. Since the proteasome activity probe assay is unable to discriminate between the activities of the various proteasome subunits, the observed changes in proteasome activity likely stem from an increased β5i subunit of the immunoproteasome, which is induced by the JAK/STAT pathway indeed [165]. Again, since C3a has been shown to downregulate the ubiquitin–proteasome pathway, despite increasing the β5i immunoproteasome subunit [38,40], a possible explanation is that the immunoproteasome is recruited to compensate for the inhibition of standard proteasome and autophagy.

An abnormal and persistent immunoproteasome activation, as it occurs during chronic inflammation, may predispose cells to cytotoxic attack by primed T-cells [177]. This is expected to include RPE cells, which upregulate MHC-I expression when exposed to the immunoproteasome-inducing cytokine interferon-gamma (IFN-γ) [178]. Intriguingly, RPE cells appear to be highly resistant to the destructive potential of primed cytotoxic T cells, due to inherent, yet still unclear mechanisms putting a brake on MHC-I expression [178]. This suggests that the immunoproteasome in RPE cells may play a role beyond antigen presentation. This is in line with an alternative stream of evidence suggesting a protective role for the immunoproteasome through enhanced clearance of oxidatively damaged proteins [179,180,181]. In fact, the genetic ablation of immunoproteasome subunits in mice hinders the ability of RPE to resist oxidative stress [179,180]. While immunoproteasome deficiency may have only minor effects on overall retinal morphology, a significant defect in retinal function is observed [182]. 

Remarkably, dysregulations of immunoproteasome expression may also variously affect autophagy by altering either AKT signaling through phosphatase and tensin homolog (PTEN) degradation or TFEB nuclear localization [174,181,183,184]. In this scenario, it appears that PTEN expression prior to injury, and thus the baseline autophagy status, is what “primes” the cell for its fate after injury. This may also involve immunoproteasome recruitment and the ability of RPE cells to resist T-cell-mediated attacks. In this context, it would be intriguing to investigate where autophagy plays a role in tuning MHC-I expression on RPE cells, since autophagy is known to surveil CD8+ T-cell response by degrading MHC-I [185]. Although it is now becoming clear that autophagy and proteasome/immunoproteasome activities are tightly intermingled, the role of immunoproteasome in RPE-AMD and its relation to the autophagy pathway remain to be elucidated. 

## 3. Lutein, Resveratrol, and *Vaccinium myrtillus* Bridging Antioxidant/Anti-Inflammatory Activity and Autophagy Activation 

Lutein and zeaxanthin and their metabolites are collectively referred to as the macular pigment (MP) or macular xanthophyll (MX), being naturally accumulated in the macula lutea region of the human retina. These compounds are obtained only through dietary sources such as green leafy vegetables and yellow and orange fruits and vegetables. Selective uptake of lutein and zeaxanthin in the MP of the retina is mediated by StARD3 and GSTP1 binding proteins, respectively [186]. The MP carotenoids filter high-intensity, short-wavelength visible light and serve as powerful antioxidants in a region being highly vulnerable to light-induced oxidative stress [186]. As reported by several clinical studies, the dietary intake of lutein/zeaxanthin is associated with a reduced risk of developing advanced AMD (either the atrophic or exudative type) [47,187,188]. In patients with atrophic AMD, lutein intake increases the macular pigment optical density (MPOD) and correlates with an increase in visual contrast sensitivity [189]. Lutein content is also reduced in the retina of AMD donor eyes compared with controls [190]. Thus, the dietary intake of lutein/zeaxanthin may prevent or even improve AMD [51,191,192,193,194,195]. As thoroughly reviewed, the potentially beneficial effects of lutein/zeaxanthin in AMD are largely based on the targeting of oxidative stress and inflammatory-related conditions which are known to increase the risk for AMD [47,51]. Instead, only a few studies focused on the effects of lutein/ zeaxanthin on cell-clearing systems. Indeed, lutein, through autophagy induction, protects human RPE cells from cell death induced by either chronic oxidative stress, staurosporine, or LED light exposure [57]. This is also correlated with a reduction in basal-VEGF release [57]. Again, lutein, through induction of autophagy, mitigates the cytotoxic effects of vital dyes indocyanine green (ICG) and brilliant blue G in human RPE cells [196]. In mice models of endotoxin-induced uveitis (EIU) and laser-induced CNV, lutein administration reduces ROS burden and the infiltration of inflammatory mediators, and it preserves rhodopsin [176,197,198]. This occurs through inhibition of STAT3 and its related pathological changes in the retina, which is reminiscent of what is reported for AT1R blockers [47,176,199]. This is intriguing, since JAK/STAT pathways and AT1R are both known to recruit the immunoproteasome [165,174,175]. On the one hand, this suggests a potential role of lutein in blunting immunoproteasome activity, which may be relevant for specific retinal conditions such as hypertensive retinopathy, wherein the immunoproteasome seems to be detrimental [174,175]. On the other hand, in A2E-containing RPE cells exposed to blue light, lutein and zeaxanthin confer cytoprotection by preventing photo-oxidation-induced impairment of the proteasome and subsequent changes in expression of MCP-1, IL-8, and CFH [58]. This supports a beneficial role of lutein and zeaxanthin through the preservation of proteasome function during persistent chronic oxidative stress. The effects of lutein on proteasome activity remain to be further investigated. 

Similar to lutein, resveratrol may produce beneficial effects in ophthalmic diseases, including AMD, due to its antioxidant, anti-inflammatory, and anti-angiogenic effects [50,200]. In AMD patients, long-term intake of resveratrol-containing supplements provides a broad bilateral improvement in ocular structure and visual function [48]. It is now quite well established that resveratrol induces autophagy through either AMPK/SIRT1 activation or PI3K/AKT/mTORC1/2 inhibition [201,202,203,204,205,206,207,208,209,210,211]. This is associated with longevity and anti-inflammatory, antioxidant, and anti-apoptotic effects, as well as lipid- and glucose-regulating effects, in a variety of experimental conditions [201,202,203,204,205,206,207,208,209,210,211]. Resveratrol-induced autophagy is also associated with the rescue of NRF-2, which is involved in autophagy-related mitophagy and mitochondriogenesis, protection from oxidative stress, and caspase-related apoptosis, as well as downregulation of NF-κB, NLRP3, and pro-inflammatory cytokines production [203,206,207,208,209,210,211]. Within RPE cells, resveratrol prevents A2E-induced mitochondrial network fragmentation [212], and it also suppresses VEGF secretion induced by inflammatory cytokines [213]. Remarkably, the administration of a resveratrol-rich formulation rescues the autophagy flux, to provide cytoprotection against either proteasome inhibition or chloroquine/hydroquinone exposure in RPE cells [59,214,215]. This is accompanied by improved mitochondrial bioenergetics, upregulated antioxidant genes, and a reduction of ROS and inflammatory molecules production [214,215]. Similar to lutein, through induction of autophagy, resveratrol also mitigates the cytotoxic effects of vital dyes indocyanine green (ICG) and brilliant blue G in ARPE-19 cells [196]. Intriguingly, resveratrol also blunts immunoproteasome activation, which is associated with autophagy induction and anti-inflammatory effects [184]. Resveratrol also meditates SIRT1-dependent downregulation of AT1R, which is associated with protection against oxidative stress and apoptosis and regulation of lipid metabolism [216]. This suggests a potential role of resveratrol in hypertensive retinopathy, where AT1R-related immunoproteasome activation occurs [174,175].

The beneficial effects of resveratrol and lutein are reproduced by *Vaccinium myrtillus* since it contains high amounts of antioxidant and anti-inflammatory polyphenols (anthocyanins and resveratrol) [49,217,218,219]. This is documented in various experimental models of retinal degeneration [217,218,219]. In subjects with AMD and dry eye, supplements containing *Vaccinium myrtillus* improve both functional and morphological parameters of the retina [220], while enhancing tear secretion and plasmatic antioxidant potential, respectively [55]. Although the effects of *Vaccinium myrtillus* on cell-clearing pathways are largely unexplored, it has been shown to protect against blue-LED-light-induced retinal photoreceptor cell damage through modulation of autophagy and inhibition of ROS production [60]. This is also associated with the downregulation of NF-κB and the pro-apoptotic proteins p38 MAPK and caspase-3/7 [60]. In fact, *Vaccinium myrtillus* mitigates inflammatory signals in various tissues, including NF-κB, inducible nitric oxide synthase (iNOS), TNF-α, IL-1β, and IL-6 [221]. In humans with metabolic syndrome, *Vaccinium myrtillus*–based dietary intervention reduces inflammation by downregulating serum high-sensitivity C-reactive protein (CRP); IL-6, IL-12, and LPS levels; and genes associated with the TLR pathway, which are implicated in AMD, as well [222]. By reducing low-grade systemic inflammation, *Vaccinium myrtillus* intake may decrease the risk of cardiometabolic diseases [222,223]. These findings suggest that *Vaccinium myrtillus,* similar to lutein and resveratrol, deserves to be further investigated concerning its potential modulation of autophagy and/or proteasome pathways. 

## 4. Conclusions and Future Perspectives

Although many additional studies are needed to confirm the effects of lutein, resveratrol, and *Vaccinium myrtillus* on autophagy and proteasome systems in AMD specifically, the few available evidences here reviewed suggest that a potential synergism between these compounds may extend to cell clearing pathways. By acting as autophagy inducers, these phytochemicals are expected to counteract the abnormal exosome secretion, which as an unconventional solution, and may lead to extracellular accumulation of potentially harmful material [224]. Nonetheless, as part of the autophagy cycle, exosome secretion may be viewed as a natural mechanism that cells have conserved to communicate with each other and share essential cell constituents within a common environment. In this context, autophagy within the RPE tunes the exosome release of components that do exert beneficial effects in the retina, including VEGF, PEDF, and αB crystallin [67,98,141,225,226]. Conversely, dysfunctional rates of cell-clearance occurring within RPE cells upon stress conditions may contribute to altering exosome content and the polarity of RPE cells, rather than solely potentiating the secretion of undigested intracellular material. In line with this, autophagy inhibition during stressful conditions in RPE cells occludes the secretion of PEDF-containing exosomes, it promotes abnormal αB crystallin secretion from the basal rather than apical RPE side, and its boosts VEGFR2 release [98,141,226]. This is in line with studies documenting an abnormal secretion of debris through exosome-engulfed material on both sides of the RPE in AMD models [26,227]. Thus, one may expect that a dysfunction in the clearance of RPE intracellular material, including oxidized/glycated proteins/lipids and inflammatory molecules, leads to the accumulation of extracellular polymorphic debris. Indeed, autophagy and proteasome markers, along with a plethora of their conventional substrates (including lipids, apoE, AGEs, beta-amyloid, alpha-synuclein, and crystallins), are detected in drusen and/or subretinal drusenoid material [26,132,148,154,155,228,229,230].

**Figure 4:**
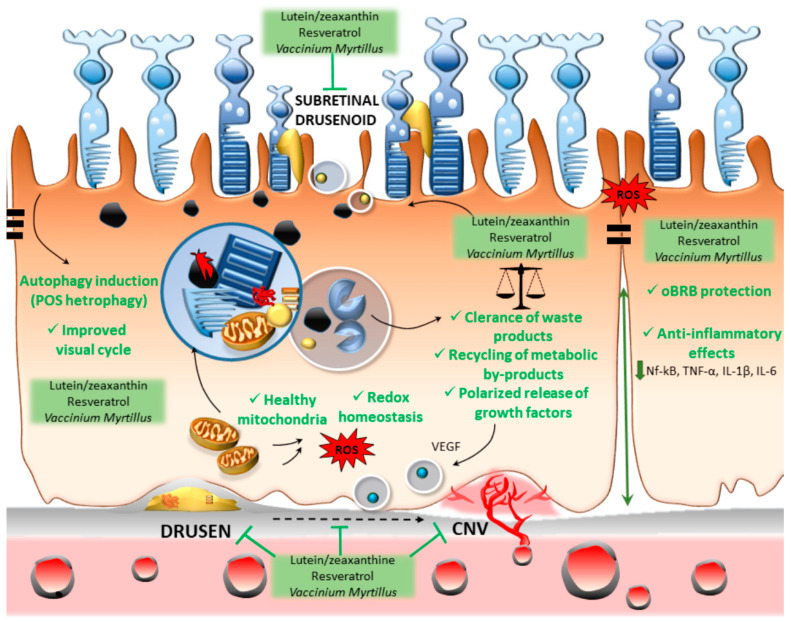
The effects of phytochemicals within the RPE. Lutein/zeaxanthin, resveratrol, and *Vaccinium myrtillus* may act through a synergistic approach involving autophagy-inducing, antioxidant, and anti-inflammatory effects. This is expected to produce an empowering of the RPE cells to metabolize properly the excess of waste substrates, while restoring the paracrine homeostasis bridging the REP with photoreceptors, the Bruch’s membrane, and the choriocapillaris. By targeting the autophagy-related dysfunction in the clearance of RPE intracellular material (including oxidized/glycated proteins/lipids, altered mitochondria, and inflammatory molecules) and by restoring the physiological RPE polarity, these phytochemicals may prevent the accumulation of extracellular polymorphic debris (drusen and subretinal drusenoid), as well as the development of choroidal neovascularization (CNV).

Altogether, these findings suggest that a failure of cell-clearing systems may underlie a loss of RPE physiological polarity to foster an abnormal bidirectional secretion, which alters retinal metabolism and function, while generating drusen and subretinal drusenoid material as a bystander effect. This calls for considering a generalized defect in substrates’ handling by the retina–choroid junction in AMD, which is best targeted by a pharmacological synergism acting at multiple levels. A phytochemical-based synergistic approach involving antioxidant, anti-inflammatory, and autophagy-inducing effects is expected to produce an empowering effect on the RPE cells to properly metabolize the excess of waste substrates, while restoring the paracrine homeostasis bridging the REP with photoreceptors, the Bruch’s membrane, and the choriocapillaris (Figure 4). In light of these considerations, we wish to conclude this manuscript by challenging the hypothesis of whether the formation of drusen and subretinal drusenoid materials, which are a classical pathological hallmark of AMD, are indeed working in the pathogenesis of AMD. Conversely, the RPE-autophagy defect per se may alter visual processing, providing the hypothetical appropriate room for beneficial effects of phytochemicals, which remain to be clearly established. Although three specific phytochemicals were here discussed as an example, we wish to point out that a much longer list of herbal compounds exists, and those compounds deserve attention for their potential beneficial effects in AMD through autophagy induction.

## Abbreviations

AMD	age-related macular degeneration
CNV	choroidal neovascularization
POS	photoreceptors outer segments
AGEs	advanced glycation end products
VEGF	vascular endothelial growth factor
VEGFR2	vascular endothelial growth factor receptor 2
oBRB	outer blood–retinal barrier
PEDF	pigment epithelium-derived factor
DHA	docosahexaenoic acid
DDC	L-dopa decarboxylase
DA	dopamine
PD	Parkinson’s disease
MAO	monoamine oxidase
EGFR	epidermal growth factor receptor
RUBCN/Rubicon	RUN domain and cysteine-rich domain-containing Beclin 1-interacting protein
mTOR	mammalian/mechanistic target of rapamycin kinase
Nrf2	nuclear factor erythroid 2-related factor 2
AMPK	5’ AMP-activated protein kinase
TFEB	transcription factor EB
ROS	reactive oxygen species
PCG1	Peroxisome Proliferator-Activated Receptor Gamma Coactivator 1-alpha
PUFAs	polyunsaturated fatty acids
A2E	N-retinylidene-N-retinylethanolamine
ECM	extracellular matrix
TXNIP	thioredoxin-interacting protein
HIF-1α	Hypoxia-Inducible Factor 1-alpha
DAMPs	danger-associated molecular patterns
NF-kB	Nuclear Factor Kappa-Light-Chain-Enhancer of Activated B Cells
TLRs	Toll-like receptors
RAGEs	advanced glycosylation end product (AGE) receptors
JAK/STAT	Janus Kinase/Signal Transducer and Activator of Transcription
NLPR3	inflammasome/NACHT, LRR, and PYD domains-containing protein 3
HSP90	heat-shock protein 90
MMP-2	matrix metalloproteinase-2
MHC-I	major histocompatibility complex I
AT1R	angiotensin II receptor type 1
IFN-	interferon-gamma
PTEN	phosphatase and tensin homolog
MPOD	macular pigment optical density
iNOS	inducible nitric oxide synthase
TNF-α	Tumor Necrosis Factor-alpha
GA	geographic atrophy
RPE	retinal pigment epithelium
